# [Retracted] Phosphoinositide 3-kinase/Akt pathway is involved in pingyangmycin-induced growth inhibition, apoptosis and reduction of invasive potential in EOMA mouse hemangioendothelioma cells

**DOI:** 10.3892/mmr.2025.13739

**Published:** 2025-11-03

**Authors:** Li-Xia Peng, Ping Zhao, Hong-Sheng Zhao, Er Pan, Bin-Bin Yang, Qin Li

Mol Med Rep 12: 8275–8281, 2015; DOI: 10.3892/mmr.2015.4447

Following the publication of the above paper, it was drawn to the Editor's attention by a concerned reader that a pair of the flow cytometic (FCM) plots featured in Figs. 1B and 4B contained areas of similarity, suggesting that these data were derived, at least in part, from the same original source. The Editorial Office also conducted an independent investigation of the data in this paper, which revealed that Transwell assay data were duplicated between Figs. 1C and 4C. Moreover, a number of the FCM plots comparing between Figs. 1B and 4B contained more extensive overlapping areas, and these FCM data had also appeared in three other papers written by different authors at different research institutes that were submitted at around the same time as this submssion to *Molecular Medicine Reports*, one of which has subsequently been retracted.

In view of the fact that the abovementioned data were published in other journals as well as *Molecular Medicine Reports*, and in view of an overall lack of confidence in the presented data, the Editor has decided that this paper should be retracted from the Journal. The authors were asked for an explanation to account for these concerns, but the Editorial Office did not receive a reply. The Editor apologizes to the readership for any inconvenience caused.

